# Long‐term follow‐up of intradetrusor botulinum toxin utilisation: A comparison of patients with multiple sclerosis and idiopathic overactive bladder

**DOI:** 10.1002/bco2.479

**Published:** 2024-12-19

**Authors:** William Chui, Joshua Kealey, Henry H. Yao, Garson Chan, Alvaro Bazo, Richard Parkinson, Helen E. O'Connell, Johan Gani

**Affiliations:** ^1^ Department of Urology, Western Health University of Melbourne Melbourne Australia; ^2^ Department of Urology, Eastern Health Melbourne Australia; ^3^ Eastern Health Clinical School Monash University Melbourne Australia; ^4^ Department of Urology Nottingham University Hospitals NHS Trust Nottingham UK

**Keywords:** bladder, detrusor, efficacy, incontinence, multiple sclerosis, neurogenic

## Abstract

**Objectives:**

To assess long‐term outcomes (up to 10 cycles) of repeated intradetrusor botulinum toxin (BoNT) utilisation in multiple sclerosis (MS) and idiopathic overactive bladder (OAB).

**Materials and Methods:**

This is a retrospective, international, multi‐centre, observational cohort study of patients diagnosed with MS and neurogenic OAB and treated with intradetrusor BoNT between January 2005 and January 2020 (just prior to COVID‐19 interruption). Dose, efficacy, duration of effect, International Consultation on Incontinence Questionnaire Overactive Bladder (ICIQ‐OAB) and International Consultation on Incontinence Questionnaire Urinary Incontinence (ICIQ‐UI) measures and complication rates were recorded. Comparisons were made to those with idiopathic OAB.

**Results:**

Seven hundred and ninety‐two patients received intradetrusor BoNT treatment (104 had MS with neurogenic OAB and 688 had idiopathic OAB). Patients with MS were more likely to receive higher doses of BoNT in all cycles. Self‐reported efficacy ranged from 85.7 to 100% (MS) and 87.2 to 100% (idiopathic) with MS patients reporting increased efficacy in cycles 1–3 comparatively (*p* < 0.05). Duration of effect ranged from 4.5 to 9 months with a reduction in the MS cohort between cycles 1 and 10 (median 8 months vs. 5 months, *p* = 0.0156).

**Conclusion:**

Patients with MS and neurogenic OAB have a good therapeutic effect from intradetrusor BoNT that is sustained over at least 10 cycles with significant reduction in the duration of action with subsequent cycles.

## 1 INTRODUCTION

Multiple sclerosis (MS) is a degenerative neurological condition often affecting people aged 55–65 with a prevalence of 300 per 100,00 people.^1^, ^2^, ^3^ It is characterised by chronic demyelination of the central nervous system leading to axonal loss.^4^ Lower urinary tract symptoms (LUTS) are a common manifestation of MS in the form of neurogenic OAB.^5^

Behavioural modification and overactive bladder (OAB) medications, such as anticholinergics or beta 3 agonists, remain first and second lines of treatments.^6^ These are used in combination with intermittent self‐catheterisation (ISC) to treat concurrent voiding dysfunction if present. MS can be a progressive disease, and LUTS and urgency incontinence often become refractory to medical therapy.^7^

Intradetrusor botulinum toxin (BoNT) A is well established in MS patients with refractory OAB.^3^ Several brands of botulinum toxin A exist. Of these, Botox®/Onabotulinum toxin A (Allergan Pharmaceuticals, Irvine, Ca, USA) and Dysport®/Abobotulinum toxin A (Ipsen biopharm Ltd, Slough, UK) have been significantly utilised in the urological setting.

BoNT is the most effective minimally invasive treatment for neurogenic OAB.^8^, ^9^ Duration of effect is approximately 6 to 9 months with significant patent variation.^10^, ^11^ Therapeutic effect can continue with repeated dosage without increasing toxicity.^12^

Previous studies have reported on the short‐term efficacy of BoNT in MS patients (up to cycle 6).^13^ Our primary aim is to report on the long‐term outcomes and response to treatment of MS patients with intradetrusor BoNT, up to cycle 10 of their treatments, to determine if efficacy and duration of effect continues with prolonged treatment. Our secondary aims are to report on the complications, dropout rates, time to follow‐up and change of treatment plans for patients with MS and neurogenic OAB treated with intradetrusor BoNT for prolonged periods and to compare these outcomes with patients with idiopathic OAB.

## 2 MATERIALS AND METHODS

This is a retrospective, international, multi‐centre, observational cohort study comparing outcomes of intradetrusor BoNT in the treatment of overactive bladder for patients with MS and neurogenic OAB versus patients with idiopathic OAB. Participating institutions were in Melbourne, Australia, and Nottingham, United Kingdom, and included public and private health care settings. Idiopathic OAB was defined as patients with OAB without any known neurogenic cause. OAB patients secondary to other neurogenic causes other than MS were excluded from this study.

Clinical data was retrospectively collected from medical records of all patients treated with intradetrusor botulinum toxin for detrusor overactivity. Dose of BoNT, efficacy, duration of effect and complication rates (including de novo urinary retention requiring ISC and UTI) were recorded. Dose and dose escalation between cycles was at clinician discretion and based on clinical symptom response. UTI was defined as symptomatic infection requiring antibiotic treatment. De novo urinary retention was defined as the need to commence ISC or have a permanent catheter placed after BoNT treatment, and this excluded patients with pre‐treatment ISC or urinary catheter. There was no absolute post‐void residual bladder volume that determined the recommendation for ISC; this was left to individual clinician and patient preference. A rising residual or poor response to BoNT related to residual volume was associated with offering of ISC education. Each patient was followed up for as many cycles of intradetrusor BoNT completed until 10 cycles were reached, until the end of the study period (January 2005–January 2020) or until decision to cease BoNT treatments was made. Our end of study period (January 2020) was chosen as that was the time before COVID‐19 started disrupting normal elective surgical lists. Ethical approval was gained from the local Human Research Ethics Committee. Due to the retrospective deidentified nature of the data, consent was not obtained.

Efficacy was defined as patients self‐reporting symptom improvement postinjection. This could be full resolution or partial resolution of symptoms. No efficacy was defined as no discernible symptom improvement upon follow‐up. Efficacy was determined by either in‐person follow‐up, phone follow‐up and/or on a global improvement scale. Improvement was quantified on a Likert scale in some centres and responses were returned via mail or brought in person to clinical reviews. Quality of life data pre‐ and post‐BoNT was established using the International Consultation on Incontinence Questionnaire Overactive Bladder (ICIQ‐OAB) and International Consultation on Incontinence Questionnaire Urinary Incontinence (ICIQ‐UI). These were posted or emailed to patients at least 4 weeks after treatment. Data on efficacy and quality of life measures were collected during follow‐up visits post‐BoNT injection.

Statistical analyses were performed using SPSS® 25 software^14^ and VassarStats website.^15^ Chi‐squared, Fisher's exact test and Mann–Whitney *U* were used to compare rates and proportions. A *p* value of less than 0.05 was considered statistically significant.

## 3 RESULTS

A total of 792 patients were included in this study. Of these, 104 (13.1%) had a diagnosis of MS with neurogenic OAB whilst 688 (86.9%) had a diagnosis of idiopathic OAB (Table 1). Urodynamic assessment was available for 444/688 (64.5%) of patients diagnosed with idiopathic OAB with 79.7% showing obvious DO. Urodynamic assessment was available for 30/104 (28.2%) of patients with MS with neurogenic OAB; of these, 76.6% showed obvious DO. Patients receiving BoNT in the MS with neurogenic OAB group were significantly younger than those in the idiopathic OAB group (median 51.5 years vs. 59 years, *p* < 0.0001). Patients in the MS cohort were more likely to be male (26.9%) compared to the idiopathic OAB group (21.2%). OnabotulinumtoxinA (Botox®) was utilised for 96.2% of patients with MS for their first injection whilst 3.8% received abobotulinumtoxinA (Dysport®). Comparatively 86.9% of patients with idiopathic OAB received Botox® whilst 13.1% received Dysport®. Dysport® use steadily decreased throughout the cycles until cycle 5 after which no patients received it (Table 2). In all cycles, patients with MS and neurogenic OAB were statistically significantly more likely to receive higher doses of BoNT than those with idiopathic OAB (Table 2).

**TABLE 1:** Patient demographics of those with multiple sclerosis and neurogenic OAB compared to those with idiopathic OAB prior to their first cycle of intradetrusor botulinum toxin A.

	MS with neurogenic OAB	Idiopathic OAB	*p* value
Number	104	688	
Age (years) (IQR)	51.5 (40.5–61.5)	59 (46–70)	<0.0001
Gender			0.20
Female	76 (73.1%)	542 (78.9%)	
Male	28 (26.9%)	146 (21.1%)	
Daytime frequency episodes (median)	9	10	
Nighttime frequency episodes (median)	2	2	
Incontinence episodes (median)	2	4	
ICIQ‐OAB	10	11	
ICIQ‐UI	14	16	
Urodynamics			0.82
No DO present	7	90	
DO present	23	354	

Abbreviations: DO, detrusor overactivity; MS, multiple sclerosis.

**TABLE 2:** Dose comparison of those with multiple sclerosis and neurogenic OAB compared to those with idiopathic OAB.

	Cycle 1		Cycle 2		Cycle 3		Cycle 4		Cycle 5	
Dose (Botox®)	Idiopathic	MS	Idiopathic	MS	Idiopathic	MS	Idiopathic	MS	Idiopathic	MS
<100 U	8	0	2	0	1	0	0	1	0	0
100 U	487	28	247	19	143	11	83	4	55	3
200 U	95	50	68	30	59	27	43	23	35	16
300 U	7	22	6	24	5	23	6	20	6	19
400 U	1	0	0	0	0	0	0	0	0	0
*p* Value		<0.001		<0.001		<0.001		<0.001		<0.001
Dose (Dysport®)										
300 U	39	0	21	0	15	0	8	0	0	0
500 U	50	1	29	1	8	1	1	0	2	0
600 U	0	0	0	0	0	1	0	0	0	0
700–800 U	1	3	2	3	0	1	0	0	0	0
*p* Value		<0.001		0.001		0.003		NA		NA

	Cycle 6		Cycle 7		Cycle 8		Cycle 9		Cycle 10	
Dose (Botox®)	Idiopathic	MS	Idiopathic	MS	Idiopathic	MS	Idiopathic	MS	Idiopathic	MS
100 U	37	1	26	0	18	0	11	0	7	0
200 U	20	11	17	9	13	5	10	4	8	2
300 U	6	17	3	17	1	16	1	13	3	10
400 U	0	2	0	0	1	0	1	0	0	2
*p* Value		<0.001		<0.001		<0.001		<0.001		<0.001

Abbreviations: MS, multiple sclerosis; U, units.

### 3.1 Dropout rates

**FIGURE 1:**
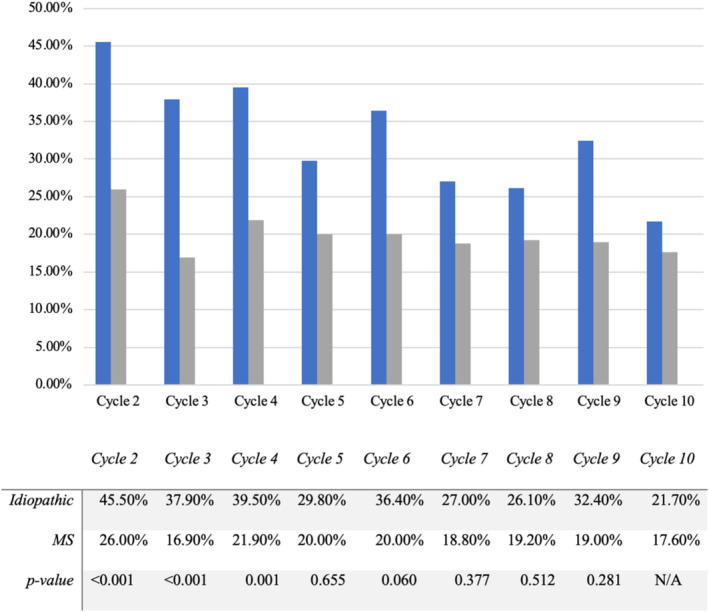
A comparison of dropout rates between patients with multiple sclerosis and neurogenic OAB compared to those with idiopathic OAB.

There was significant dropout in each cycle for both the idiopathic OAB and MS groups that ranged between 21.7 and 45.5% and 16.9 and 26.0%, respectively, for each cycle. Dropout rates for both groups were generally higher in the early cycles and lower in the later cycles (Figure 1). In all cycles, the dropout rates for the idiopathic OAB group were higher than the MS group. MS patients were thus more likely to continue treatment as cycles progressed. This was statistically significant in cycles 2, 3 and 4 (*p* < 0.05).

### 3.2 Efficacy and duration of effect

Self‐reported efficacy post‐BoNT injection ranged from 85.7 to 100% for patients with MS and 87.2 to 100% for patients with idiopathic OAB. MS patients were statistically significantly more likely to report efficacy from their BoNT injections in cycles 1–3 compared to patients with idiopathic OAB. As progressive cycles continued there was no statistically significant difference between groups (Table 3). Median duration of effect ranged from 4.5 to 9 months in the MS group and 5.5 to 8 months in the idiopathic OAB group for each cycle with no aetiology showing a sustained prolonged duration compared to the other. However, duration of effect decreased over time in both MS patient and idiopathic OAB groups (Table 3). The MS patient cohort had a median of 8 months in the first cycle compared to 5 months in cycle 10 (*p* = 0.0156) whilst the idiopathic OAB cohort had a median of 7 months in the first cycle compared to 6 months in cycle 10 (*p* = 0.0107) (Table 3).

**TABLE 3:** A comparison of duration of effect and efficacy from intradetrusor botulinum toxin between those with multiple sclerosis and neurogenic overactive bladder compared to those with idiopathic overactive bladder.

	Cycle 1	Cycle 2	Cycle 3	Cycle 4	Cycle 5	Cycle 6	Cycle 7	Cycle 8	Cycle 9	Cycle 10
Duration										
MS	8 (6–11)	9 (7–12)	8.5 (6–12)	4.5 (6–8)	6 (5–9)	6 (3–7)	7 (6–9)	6 (3–6)	4.5 (4–6)	5 (4–6)
Idiopathic	7 (6–10)	8 (6–11)	8 (6–11)	6 (8–10)	7 (5–10)	6 (5–10)	6 (5–6)	5.5 (6–9)	6 (5–6)	6 (5–8)
*p* Value	0.2403	0.1383	0.4125	0.0245	0.4423	0.1982	0.0314	0.0819	0.018	0.1107
Duration over time (vs. cycle 1)										
MS (*p* value)	NA	0.6223	0.9526	0.0276	0.0816	0.0587	0.0312	0.0703	0.0020	0.0156
Idiopathic (*p* value)	NA	0.6583	0.1878	0.0495	0.0206	0.2874	0.5758	0.0266	0.0126	0.0107
Efficacy										
MS	96.7%	100%	100%	94.6%	96.2%	100%	100%	92.3%	85.7%	100%
Idiopathic	87.2%	90%	89.9%	93.2%	91.3%	89.8%	94.1%	89.3%	94.4%	100%
p Value	0.007	0.004	0.015	1	0.669	0.311	0.531	1	0.568	NA

Abbreviations: MS, Multiple Sclerosis, UTI, Urinary Tract Infection.

In both MS and idiopathic patient groups, there was an increasing proportion of patients needing higher doses of BoNT (≥ 300 U) with subsequent cycles. This appeared more pronounced in MS patients. In the MS cohort, there was a statistically significant difference (*p* < 0.05) in the proportion of patients requiring ≥ 300 U of BoNT all throughout cycles 3 to 10 (Table 4). Conversely, for the idiopathic group this was statistically significant in cycles 4, 5, 6, 9 and 10.

**TABLE 4:** Proportion of patients needing higher doses (≥ 300 U) of intradetrusor botulinum toxin with subsequent cycles when compared to cycle 1.

	Cycle 2	Cycle 3	Cycle 4	Cycle 5	Cycle 6	Cycle 7	Cycle 8	Cycle 9	Cycle 10
MS									
<300 U	49	38	28	19	12	9	5	4	2
≥300 U	24	23	20	19	19	17	16	13	12
*p* Value	0.110	0.031	0.013	0.001	0.003	<0.001	<0.001	<0.001	<0.001
Idiopathic									
<300 U	317	203	126	90	57	43	31	21	15
≥300 U	6	5	6	6	6	3	2	2	3
*p* Value	0.578	0.338	0.027	0.007	0.019	0.038	0.092	0.049	0.003

Abbreviations: MS, multiple sclerosis; U, units.

### 3.3 Complications

UTI rates ranged between 4.8 and 23.8% in patients with MS and 0–4.5% in patients with idiopathic OAB for each cycle (Table 5). Several cycles showed patients with MS were significantly more likely to develop a UTI post‐injection compared to patients with idiopathic OAB (cycle 1, *p* = 0.002; cycle 3, *p* = 0.02; cycle 5, *p* = 0.006; cycle 6, *p* = 0.002). De novo urinary retention occurred in MS patients who underwent BoNT in 44.1%, 44.0% and 25.0% of patients in cycles 1, 2 and 3, respectively; however, this ceased after cycle 3. This is in comparison to idiopathic OAB patients who had a more consistent rate of de novo urinary retention throughout all cycles (Table 5).

**TABLE 5:** A comparison of complications from intradetrusor botulinum toxin between those with multiple sclerosis and neurogenic overactive bladder compared to those with idiopathic overactive bladder.

	Cycle 1	Cycle 2	Cycle 3	Cycle 4	Cycle 5	Cycle 6	Cycle 7	Cycle 8	Cycle 9	Cycle 10
UTI										
MS	12.40%	4.80%	8.00%	8.10%	17.90%	23.80%	5.60%	6.30%	14.30%	7.70%
Idiopathic	4.0%	4.40%	1.10%	4.50%	1.40%	0%	0%	0%	0%	0.00%
*p* Value	0.002	0.748	0.02	0.411	0.006	0.002	0.367	0.348	0.172	0.481
De novo retention										
MS	44.10%	44.00%	25.00%	0%	0%	0%	0%	0%	0%	0%
Idiopathic	31.80%	23.60%	22.70%	18.00%	24.60%	18.90%	17.40%	15.00%	20%	18.20%
*p* Value	0.096	0.026	1	0.585	1	1	1	NA	NA	NA

Abbreviations: MS, multiple sclerosis; UTI, urinary tract infection.

### 3.4 Quality of life measures

**FIGURE 2:**
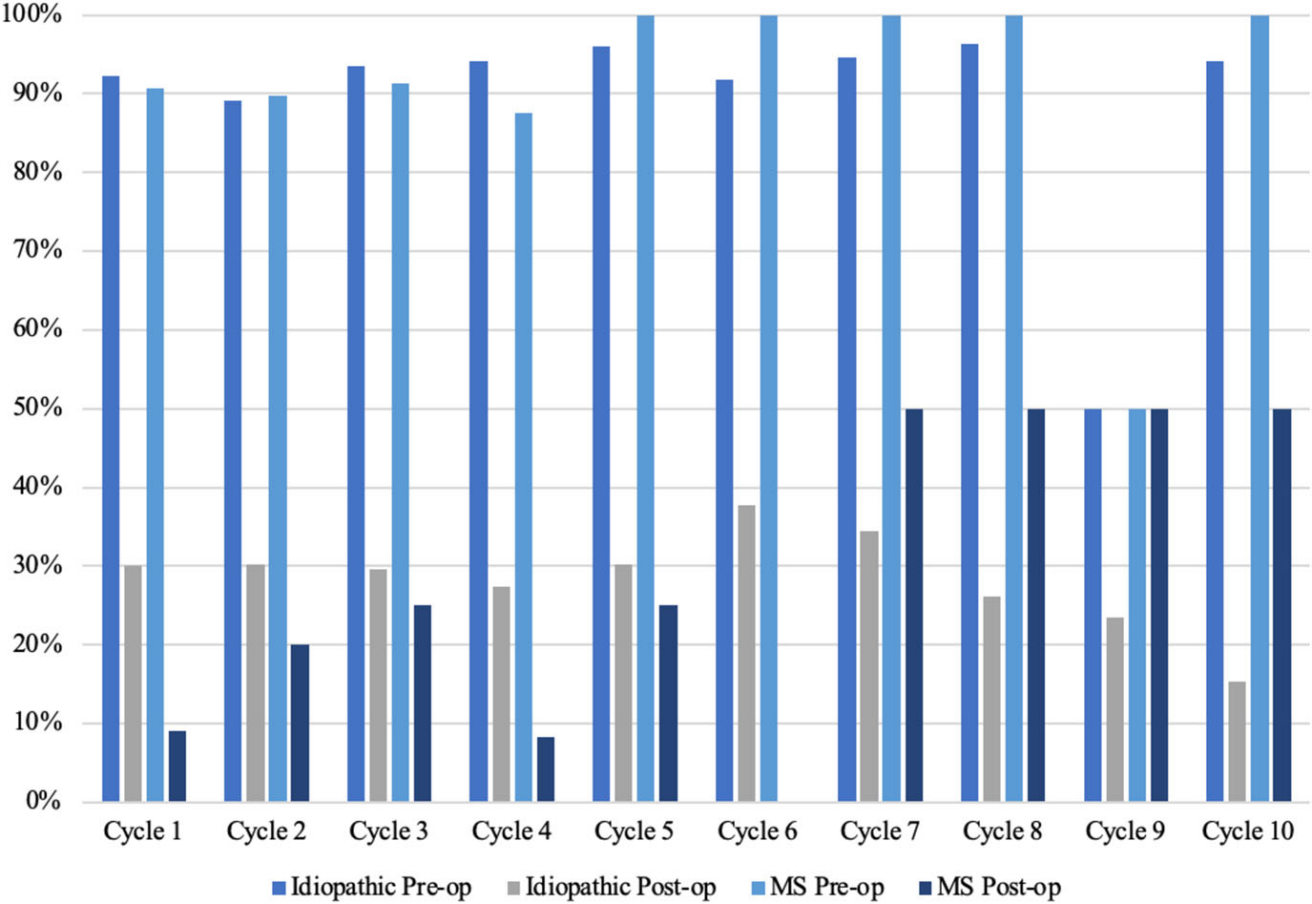
A comparison of incontinence rates pre‐ and post‐intradetrusor botulinum toxin injection between patients with multiple sclerosis and neurogenic OAB versus those with idiopathic OAB.

Pre‐operative incontinence rates ranged between 50.0 and 96.6% in the idiopathic OAB cohort and 50 and 100% in the MS cohort for each cycle (Figure 2). Post‐operative incontinence rates improved significantly and ranged from 15.4 to 37.8% in the IDO cohort and 0 to 50% in the MS cohort for each cycle. Improvement in incontinence rates was maintained throughout all cycles for both groups. In cycle 1, patients with MS were statistically significantly more likely to regain continence than those with idiopathic OAB (*p* = 0.008); however, this was not replicated in further cycles. Each cycle displayed a significant improvement in incontinence episodes for both MS and idiopathic OAB patients which was maintained throughout all 10 cycles.

Median pre‐operative ICIQ‐OAB scores ranged from 3.5 to 10 in the MS group and 9 to 11 in the idiopathic OAB group, whilst ICIQ‐UI scores ranged from 10.5 to 14 in the MS group and 14 to 16 in the IDO group (Table 6). Median post‐operative ICIQ‐OAB scores improved significantly in both groups to range from 0 to 2 in the MS cohort and 1 to 3 in the idiopathic OAB cohort as did the median post‐operative ICIQ‐UI scores to range from 0 to 5.5 in the MS group and 0 to 3 in the idiopathic OAB cohort.

**TABLE 6:** A comparison of the change in ICIQ‐OAB, ICIQ‐UI, daytime frequency, nocturnal frequency and incontinence episodes pre‐ and post‐intradetrusor botulinum toxin injection in those with multiple sclerosis and neurogenic OAB and those with idiopathic OAB.

		Change in ICIQ OAB	Change in ICIQ UI	Change in daytime frequency	Change in nocturnal frequency	Change in incontinence episodes
Cycle 1						
Idiopathic	*N*	311	314	350	349	346
	Median (IQR)	7 (4–10)	11 (5–15)	4 (2–7)	1 (0–2)	3 (1–5)
MS	*N*	31	31	33	33	33
	Median (IQR)	7 (6–12)	12 (7–15)	4 (3–6)	1 (0–2)	2 (1–6)
Cycle 2						
Idiopathic	*N*	145	147	169	171	172
	Median (IQR)	6 (3–8)	9 (5–14)	3 (2–5)	1 (0–2)	2 (1–4)
MS	*N*	17	17	20	20	20
	Median (IQR)	7 (5–8)	11 (8–15)	3 (1.5–4)	1 (0–2)	3 (1–5)
Cycle 3						
Idiopathic	*N*	90	92	102	103	107
	Median (IQR)	6 (3–8)	9.5 (5–13)	3 (1–5)	1 (1–2)	2 (1–4)
MS	*N*	15	16	16	16	16
	Median (IQR)	4 (3–5)	9.5 (5–13)	1.5 (1–3)	1 (0–2)	3 (1–5.5)
Cycle 4						
Idiopathic	*N*	67	69	71	71	72
	Median (IQR)	5 (2–9)	10 (5–13)	3(1–6)	1 (0–2)	3 (1–4)
MS	*N*	11	11	12	12	12
	Median (IQR)	5 (2–9)	13(7–14)	1.5 (0–4)	0 (0–1.5)	2 (0.75–3)
Cycle 5						
Idiopathic	*N*	46	46	52	52	52
	Median (IQR)	7 (5–9)	12(7–16)	2(1–4.5)	1 (0–2)	3 (1–5)
MS	*N*	6	6	8	7	8
	Median (IQR)	5.5 (4–7)	13.5 (7–16)	3.5 (1–9.5)	1 ()0–3)	2 (0.75–3)
Cycle 6						
Idiopathic	*N*	34	34	38	37	37
	Median (IQR)	7 (3–10)	10.5 (4–17)	3(1–4)	1 (0–2)	2 (1–4)
MS	*N*	5	5	5	5	5
	Median (IQR)	7 (7–7)	10 (10–14)	2(0–6)	1 (0–1)	2 (2–3)
Cycle 7						
Idiopathic	*N*	28	28	29	29	28
	Median (IQR)	7 (5.5–9)	11.5 (8–15.5)	3 (1–4)	1 (1–2)	3 (1.25–4)
MS	*N*	3	2	3	3	4
	Median (IQR)	7 (1–9)	8.5 (4–13)	2 (0–8)	1 (0–2)	2.75 (1–3.75)
Cycle 8						
Idiopathic	*N*	22	21	22	22	22
	Median (IQR)	8 (5–9)	12 (8–18)	3.5 (1–5)	1 (0–2)	3 (2–6)
MS	*N*	2	2	2	2	2
	Median (IQR)	6 (6–6)	4.5 (3–6)	3.5 (2–5)	1.5 (1–2)	2 (2–2)
Cycle 9						
Idiopathic	*N*	17	17	17	17	17
	Median (IQR)	9 (6–10)	11 (8–17)	4 (2–6)	1 (0–1)	3 (1–4)
MS	*N*	2	2	2	2	2
	Median (IQR)	2.5 (2–3)	9 (8–10)	2 (0–4)	0.5 (0–1)	1.5 (1–2)
Cycle 10						
Idiopathic	*N*	10	10	11	11	12
	Median (IQR)	9 (7–11)	14.5 (10–18)	5 (2–6)	1 (1–2)	5.5 (2–9)
MS	*N*	2	2	2	1	2
	Median (IQR)	4 (2–6)	7.5 (5–10)	3.5 (2–5)	0 (0–0)	2.5 (2–3)

Abbreviations: ICIQ‐OAB, International Consultation on Incontinence Questionnaire Overactive Bladder Module, ICIQ‐UI, International Consultation on Incontinence Questionnaire Urinary Incontinence; MS, multiple sclerosis.

Median pre‐operative day time frequency episodes ranged from 6 to 9 in the MS group and 7 to 10 in the idiopathic OAB group, whilst nighttime episodes ranged from 0 to 2 in the MS group and was 2 in the idiopathic OAB group. Daytime episodes improved significantly ranging from 4 to 5, in both groups. Nighttime episode also improved in both groups to 0 in the MS group and 1 in the idiopathic OAB group. Pre‐operative incontinence episodes improved from a median of 3–4 to 0 in the idiopathic OAB group and a median of 2–4 to 1 in the MS group. These improvements in ICIQ‐OAB, ICIQ‐UI, frequency episodes and incontinence episodes were sustained throughout the 10 cycles in both groups. The change in daily incontinence episodes was significant in both groups and improved from a pre‐operative median of 3–4 to 0 in almost all cycles.

There was no significant difference between idiopathic OAB and MS patients in the change in pre‐ and post‐operative ICIQ‐OAB, ICIQ‐UI, daytime or nighttime frequency or incontinence episodes.

## 4 DISCUSSION

Our multicentre, international study is the largest analysis of long‐term efficacy and safety of MS with neurogenic OAB and idiopathic OAB patients who underwent BoNT. Previous studies have analysed shorter term outcomes, especially in the MS with neurogenic OAB cohort.^16^, ^17^ This study demonstrated patients with MS with neurogenic OAB have good therapeutic effect from intradetrusor BoNT that is sustained with progressive cycles. This was established via subjective efficacy, improved continence, urgency episodes (day and night), OAB questionnaire scores and incontinence episodes. This is consistent with other studies that have investigated the therapeutic effect in MS with neurogenic OAB patients, but on a larger scale and through more prolonged cycles.^16^, ^17^, ^18^ The findings of improved efficacy in early cycles with a reduction in duration of effect in later cycles in the MS with neurogenic OAB cohort comparatively has not previously been reported. This may form an important aspect of counselling these patients prior to BoNT treatment.

MS patients reported higher rates of efficacy than those with idiopathic OAB in early cycles, which could account for the lower dropout rate in the MS cohort per cycle. This may be due to statistically significantly higher doses of BoNT used from the outset in the MS cohort. Whilst higher doses of BoNT in MS patients are suggested in guidelines, there is increasing evidence that low dose (100 U) may be adequate for significant therapeutic effect and lower rates of urinary retention.^19^ Initial dosing for idiopathic OAB patients was mostly 100 U; however, some of these patients received higher doses which may suggest differing regional practices or patients who received their first dose of BoNT prior to established 100 U guidelines. Although efficacy was maintained in the MS with neurogenic OAB cohort, there was a significant reduction in the duration of effect as cycles progressed from cycles 1 to 10 (8 vs 5 months, *p* = 0.0156) which could be attributed to the progressive nature of MS.

Dropout rates were higher in the idiopathic OAB cohort in nearly all cycles. Both cohorts displayed higher dropout in earlier cycles than in later cycles, 45.5% in cycle 1 vs 21.7% in cycle 10 in the idiopathic OAB cohort and 25.0% in cycle 1 vs 17.6% in cycle 10 in the MS cohort. The dropout rates in this study are higher than in other smaller published retrospective studies.^20^, ^21^ Explanations for this could include primary and secondary failure, progression of neurological disease, patient preference and improvement in urinary continence not related to BoNT.

Rates of UTI were significantly higher in the MS with neurogenic OAB cohort in this study and reflected rates in smaller studies of patients with neurogenic OAB.^22^ There are several potential underlying factors that predispose patients with MS and neurogenic OAB to UTI including higher post‐void residual volumes secondary to concomitant voiding dysfunction that are exacerbated by BoNT, and a higher proportion of patients doing ISC.

Both groups showed higher than previously reported rate of de novo urinary retention post‐BoNT. Higher rates of retention may have occurred from higher doses of BoNT in both cohorts at first dose. The MS cohort showed significantly higher rates in early cycles (25 to 44.1% in the first three cycles) compared to those with idiopathic OAB. These high rates of retention may be attributed to the use of Dysport®. Ravindra et al. had found that Dysport® had higher rates of urinary retention when compared to Botox® (42% vs. 23%) in idiopathic OAB.^23^

Quality of life measures displayed strong, sustained improvements in both the MS and IDO groups. MS patients tended to have lower pre‐operative ICIQ‐OAB and ICIQ‐UI scores than the IDO cohort. A potential explanation for this is more significant impairment in other domains, such as mobility and fatigue, that had more severe impact on quality of life comparatively. MS patients also generally reported a similar number of pre‐operative daytime and nighttime frequency episodes as the IDO group. When comparing the change in pre‐ and post‐operative scores, however, there was no significant difference between groups to suggest one cohort improved more than the other with treatment.

Patients in this study with MS with neurogenic OAB were younger than those treated for idiopathic OAB and were less likely to have undergone urodynamic assessment. MS patients were statistically significantly more likely to receive higher doses of intradetrusor BoNT across all cycles than those with idiopathic OAB. There was a larger proportion of males in the MS group than the idiopathic OAB group which also reflects the population affected by MS compared to idiopathic OAB. The utilisation of both Botox® and Dysport® further allows for the generalisability of this study when treating patients with intradetrusor BoNT.

There are several limitations to this study. The retrospective nature of this study relied on accurate and timely medical records. These were heterogeneous between practices and relied on patient recall for some aspects. Non‐responders to BoNT and those with adverse effects are more likely to be lost to follow‐up or further treatments as cycles progress that may also confound results. Response rates for questionnaires were relatively low in both groups which could introduce potential bias when assessing improvements from the questionnaires. There was no way to determine which patients were lost to follow‐up. Some patients who were assumed to dropout of ongoing BoNT treatments may have had a prolonged delay to further treatments and not truly dropped out. There was no way to separate these patients at time of data review; however, the number is expected to be small and equal among cohorts. There were also low urodynamic rates in MS patients. This can be attributed to the retrospective nature of the study whereby not all pre‐operative data was available. In addition, there could have been a subset of MS patients that did have urodynamics performed, but clinical documentation on this was not available to be collected. Additionally, it is also likely that in the public health system, there are limited urodynamic theatre list spots. Therefore, some patients may have progressed directly to intradetrusor BoNT treatment without prior urodynamics.

## 5 CONCLUSION

This study showed that when compared to patients with idiopathic OAB, MS patients with neurogenic OAB patients demonstrate similar beneficial therapeutic effects from intradetrusor BoNT that is sustained over at least 10 cycles. In the earlier cycles from cycles 1 to 3, MS patients were more likely to report increased efficacy. Whilst efficacy was maintained throughout all cycles, there was significant reduction in the duration of action with subsequent cycles in both groups and a need for higher doses of BoNT administration.

## AUTHOR CONTRIBUTIONS

William Chui: writing—original draft, writing—review and editing, formal analysis. Joshua Kealey: writing—review and editing, investigation. Henry H. Yao: writing—review and editing, formal analysis, investigation. Garson Chan: writing—review and editing, investigation. Alvaro Bazo: writing—review and editing, investigation. Richard Parkinsons: writing—review and editing, supervision, conceptualisation. Helen E. O'Connell: writing—review and editing supervision, conceptualisation. Johan Gani: writing—review and editing, supervision, conceptualisation.

## CONFLICT OF INTEREST STATEMENT

The authors declare that there is no conflict of interest.
